# Oral Health-Related Quality of Life Among Narcotic and Stimulant Users Referred to Maintenance Methadone Therapy Centers in Ahvaz City: Iran

**DOI:** 10.3389/fpubh.2022.850550

**Published:** 2022-05-20

**Authors:** Fatemeh Saki, Maria Cheraghi, Hashem Mohamadian, Fataneh Ghorbanyjavadpour

**Affiliations:** ^1^Department of Community Oral Health, School of Dentistry, Ahvaz Jundishapur University of Medical Sciences, Ahvaz, Iran; ^2^Social Determinant of Health Research Center, Department of Community Oral Health, School of Dentistry, Ahvaz Jundishapur University of Medical Sciences, Ahvaz, Iran; ^3^Department of Health Education and Promotion, School of Health, Ahvaz Jundishapur University of Medical Sciences, Ahvaz, Iran; ^4^Department of Orthodontics, School of Dentistry, Ahvaz Jundishapur University of Medical Sciences, Ahvaz, Iran

**Keywords:** quality of life related to oral health, addict, narcotics, stimulants, quality of life

## Abstract

**Introduction:**

We aimed to assess quality of life related to oral health in narcotic or stimulant users those were referred to maintenance methadone therapy (MMT) centers in Ahvaz City, Iran.

**Methods:**

It was a cross-sectional study based on exploratory approach which has conducted on 187 narcotic and stimulant users in Ahvaz city; during 15th May till September 2020. Data was selected by available non-random sampling method. The data collection tools included the demographic variables and the standard OHIP-14 questionnaires. All tests were used as descriptive statistics, Kolmogorov-Smirnov tests, independent *t*-test, one-way analysis of variance. *P*-values of less than 0.05 was considered significant.

**Results:**

The mean and standard deviation of the participants' age was 36.03 ± 8.98 years. The quality-of-life scores related to oral health were totally 34.89 ± 6.50 as well as 37.37 and 33.96 in narcotic and stimulant users, respectively. The total quality of life related to OHIP-14 did not have a significant relationship with variables of age, life companions, level of education, number of children, economic status, employment status, insurance status, underlying disease, toothbrush use status, last dentist visit, and number of missing teeth (*P* > 0.05). However, a significant difference was found between the quality of life related to oral health based on the type of substance used (narcotic or stimulant), so that the mean quality of life related to oral health was higher in narcotic than stimulant users (*P* < 0.05).

**Conclusion:**

Quality of life related to OHIP-14 was more unfavorable in stimulant users than narcotic users. So, policy makers and authorities are required to focus their interventions and research programs to improve health-related quality of life in users, especially stimulant.

## Introduction

Use of addictive substances is among the most challenging and complex health problems leading to a wide range of cardiovascular, mental and psychological, metabolic, endocrine, and infectious disorders (1). Addiction to new drugs and industrial substances not only gives rise to many social and economic problems, but also causes damage to oral health (2). Opioids, as the most commonly used drug in Iran, include opium, syrup, heroin, and codeine used orally, by inhalation, or by injection. Other substances used in Iran include cannabis (from the cannabis group), stimulants such as methylphenidate (under the brand name of Ritalin from the amphetamine group), ecstasy (from the amphetamine group), as well as cocaine and LSD (from the hallucinogenic group) (2). The use of narcotics or stimulants causes dry mouth or xerostomia, which in turn reduces the saliva pH and increases the formation of plaque and dental plaque. All these factors increase the incidence of tooth decay and periodontal disease (1). Moreover, narcotic and stimulant addiction can increase the tendency to intake simple sugars, which is an effective factor in causing caries and bruxism and increasing tooth sensitivity and necrotic gingivitis (3). Given that narcotic or stimulant addiction reduces motivation and self-confidence, addicts and those treated with methadone have lower rates of oral hygiene than non-addicts (4). Mental problems such as depression caused by drug addiction can eventually lead to neglect oral care and follow-up of dental treatment except in emergencies (5, 6).

Health-related quality of life is specifically associated with quality of life in relation to health and disease, which is a multidimensional concept. Oral health quality of life is one of these dimensions since oral diseases have been proven to affect the quality of life (1). Oral health-related quality of life is defined as an individual's personal assessment of the effects of functional, physical, and social factors as well as experience of oral pain and discomfort on different aspects of their life (7). Given the role that oral conditions play in people's social relations, appearance, self-confidence, as well as speaking, chewing, tasting, and swallowing processes, they affect one's pleasure experienced from life. Therefore, the status of oral health-related quality of life affects people's lives in both physical and mental dimensions (8).

The seriousness and importance of dental problems in treating substance abusers necessitate establishment of an accessible dental care program. For example, the toothache interfering with the process of addiction treatment may cause relapse in addicts. In addition, poor appearance and improper functioning of the dental problems can cause isolation and non-compliance with the treatment and care process (9).

So far, few studies were conducted on oral health and its related quality of life among the addicts worldwide (10–12) and in Iranian cities of Yazd, Mashhad, Isfahan, and Tehran (13–19). Some of these studies concluded that drug abuse increased DMFT and Plaque Control Record indices (14). As they noted, stimulants and morphine were the most commonly used drug among women and men, respectively (16). Furthermore, the oral and dental treatment needs of the addicted population are wide and should be followed up (14, 15). Akbari et al. (13) also reported an urgent need to plan dental treatments in the population of addicts at the time of withdrawal. In this regard and since no research has ever investigated oral health status related to narcotic and stimulant abuse in Ahvaz City, the present study was conducted. The aim was to determine the quality of life related to oral health of narcotic and stimulant addicts who referred to maintenance methadone therapy (MMT) centers in Ahvaz City in 2020.

## Methods

### Participants and Methodology

It was a cross-sectional study based on exploratory approach which has carried out among 187 narcotic and stimulants users who referred to MMT centers using available non-random sampling method in Ahvaz City during 15th May till September 2020. The cluster sampling method was conducted from the MMT centers in the east and west of Ahvaz. From each cluster, nine centers were randomly selected. In each center, available sampling method was applied and eligible patients who referred to the MMT centers entered the study. Initially, the participants were explained about the research goal and process. Followed by obtaining informed consent forms, the researcher completed the questionnaires for each participant. Inclusion criteria were having satisfaction to participate in the study, 18 years of age and older, as well as ability to communicate and answer the questions. Exclusion criteria included unwillingness to continue cooperation at any stage of the study and incomplete questionnaires. The participants provided answers to the questionnaire items, which were facilitated or completed by the researcher or the patient's companions if necessary.

### Sample Size

We had used to calculation of sample size by software of G-power base on exploratory *post hoc* approach with 95% confidence interval and Study accuracy (d = 0.05), power 85%, Zoc / 2 = 96.1 and effect size: 0.5 was 187.

### Inclusion Criteria

➢ People with Narcotic and Stimulant users who were referred to Methadotherapy centers in Ahvaz city during 2020.
➢ They are consent to participate in the study.
➢ Aged more than 18
➢ They had ability to speak in Persian

### Exclusion Criteria

➢ Dissatisfaction to participating in the study
➢ Failure to complete the questionnaire

### Study Tools

The study tools included a questionnaire of demographic and contextual variables containing information such as age, gender, education, marriage, number of children, employment, life partners, economic status, insurance status, underlying disease, toothbrush and toothpaste use status, last dentist visit, and number of missing teeth. Furthermore, the standard questionnaire of quality of life related to OHIP-14 was administrated, which was designed to measure the effects of oral disorders on people's health based on the respondents' self-judgment. This questionnaire was translated into Persian by Motallebnejad et al. (20). The respondents are required to answer the items on a five-point Likert scale (Never = 1, Rarely = 2, Sometimes = 3, Almost always = 4, and Always = 5). This questionnaire investigates seven dimensions of functional limitation, physical pain, mental distress, physical disability, mental disability, social disability, and physical disability. The minimum and maximum attainable scores of the questionnaire are 14 and 70, respectively, so that greater scores indicate higher quality of life related to oral health (20). In the study by Navabi et al. (21), Cronbach's alpha coefficient of the questionnaire was 0.809. In the present study, Cronbach's alpha coefficient was calculated and showed a good result for all dimensions (Functional limitations 0.883, Physiological Pain 0.874, Mental Distress 0.924, Physiological Disability 0.937, Psychological Disability 0.935, Social Disability 0. 955, Handicap 0.945 and total 0.983. Validity of instrumentation with the exploratory agent approach was acceptable for the opioid group 70.167 and for the stimulant group 61.862.

### Ethical Considerations

In order to observe ethical considerations, participants were ensured about confidentiality of information. To this end, all questionnaires were coded. Moreover, the participants were clearly explained about the study objectives and finally, those who were willing to participate in the study were surveyed. The study protocol, approved by the Ethics Committee of Ahvaz Jundishapur University of Medical Sciences, was registered with the ethics code of IR.AJUMS.REC.1399.808.

### Data Analysis

To describe and analyze the data, IBM SPSS Statistics for Windows, Version 24.0. Armonk, NY: IBM Corp was run. Descriptive statistics were applied to describe characteristics of the participants, central indices, mean dispersion, and standard deviation were used. Kolmogorov-Smirnov test was also applied to evaluate distribution of data, while independent *t*-test and one-way analysis of variance were run to determine the relationship between variables.

## Results

The mean age of addicts who referred to addiction treatment centers in Ahvaz was 36.03 ± 8.98 years and 97 addicts (52%) were under 36 years old. In terms of using toothbrush, 128 addicts (68%) rarely brushed their teeth, 127 (67.9%) did not have any dental visits for more than 3 years, and 86 addicts (46.1%) had more than five teeth lost. Table 1 represents other demographic characteristics of the participants along with the relationship of these characteristics with the mean quality of life related to oral health.

**Table 1:** Relationship between quality of life related to oral health and variables of study.

**Demographic characteristics**		**Percent**	**Quality of life mean scores related to oral health**	***P*-Value**
Age	<36 years old	51.8	34.86	0.211
	>36 years old	48.2	34.92	
Educational level	Less than a diploma	34.2	38	0.424
	Diploma or Bachelore's degree	54.4	34.67	
	Higher than Bachelore's degree	11.3	35.10	
Life companions	Only with spouse	17.1	36.84	0.208
	Only with children	9.6	34.83	
	With spouse and children	36.4	33.88	
	Alone	9.6	35.05	
	Others	27.3	34.98	
Economic status	Poor	44.3	34.87	0.137
	Moderate	44.3	34.36	
	Good	9.6	36.33	
	Excellent	1.8	33.33	
Background diseases	Yes	28.3	33.90	0.181
	No	71.7	35.26	
Occupational status	Employed	29.5	34.27	0.194
	Retired	16	34.83	
	Unemployed	54.5	35.37	
Using tooth brush	Every other day	16	36.36	0.249
	Once in a week	37.9	37	
	Rarely	46.1	35.86	
Dentist visit	In the last year	16	35.14	0.183
	In the last 1–3 years	37.9	34.75	
	>3 years	46.1	34.87	
Lost teeth	Less than 3 teeth	16	32.60	0.292
	1–3 teeth	37.9	34.85	
	More than 3 teeth	46.1	35.72	

Table 1 shows that the mean scores of qualities of life related to oral health were not significantly different between levels of study factors such as age, educational level, employment status, background disease, economic status, dental visit, use of toothbrush or number of lost teeth (*P* < 0.05).

The mean scores of qualities of life related to oral health and its dimensions were also determined based on the type of substance used; i.e., narcotics or stimulants (Table 2).

**Table 2:** Mean scores of qualities of life related to oral health of addicts wise.

**Quality of life related to oral health**	**Stimulants**		**Narcotics**		**Total**		**Significance level**
	**Mean**	**Std. deviation**	**Mean**	**Std. deviation**	**Mean**	**Std. deviation**	
Functional limitation	4.86	1.80	4.88	1.90	4.86	1.74	0.942
Physical pain	4.91	1.85	5.43	1.99	5.05	1.82	0.100
Mental distress	5.65	1.81	6.63	1.84	5.91	1.89	<0.0001
Physical disability	4.75	1.99	5.27	2.08	4.84	1.87	0.116
Mental disability	4.48	1.86	5.49	1.66	4.75	2.02	<0.0001
Social disability	4.51	1.84	4.21	1.43	4.43	1.86	0.299
Physical disability	4.77	1.60	5.45	2.00	4.96	1.74	0.019
Total	33.96	6.20	37.37	6.70	34.89	6.50	<0.0001
Number (%)	136	72.7	51	27.3	187	100	

The results obtained from Table 2 show a statistically significant difference between the quality of life related to oral health based on the type of substance used (narcotic or stimulant drugs) (*P* < 0.05). In other words, the mean quality of life related to oral health was higher in narcotic addicts than stimulant users. Moreover, a significant difference was found between narcotic and stimulant users with regard to the scores of mental distresses (*P* = 0.001), mental disability (*P* = 0.001), and physical disability (*P* = 0.019) dimensions.

**Figure 1:**
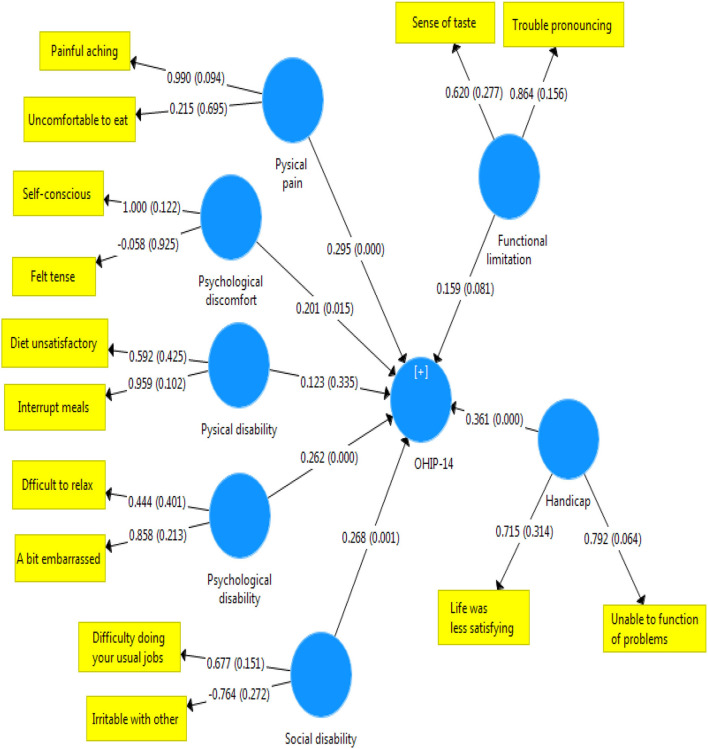
Status and severity of the relationship between the scores in the dimensions of OHIP-14 scale and the total score of quality of life related to oral health in stimulant addicts.

**Figure 2:**
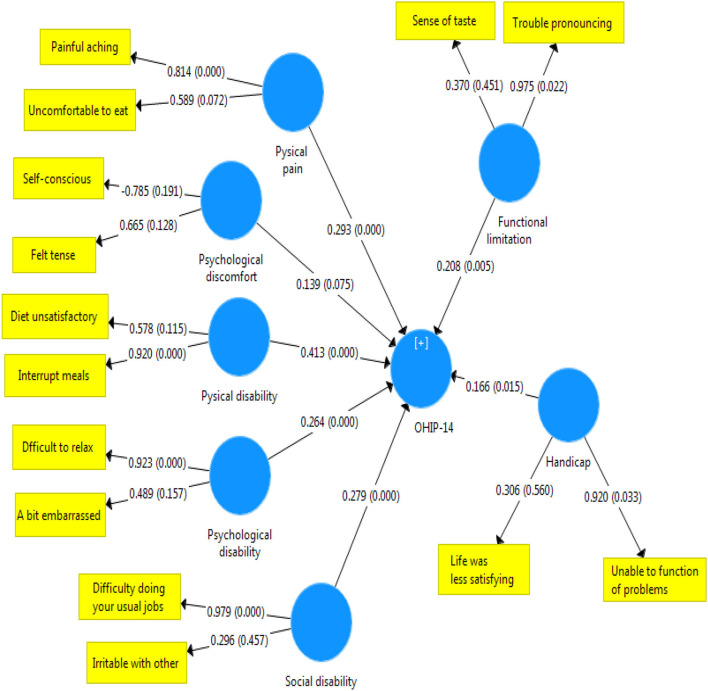
Status and severity of the relationship between scores in the dimensions of OHIP-14 scale and the total score of quality of life related to oral health in narcotic user.

**Figure 3:**
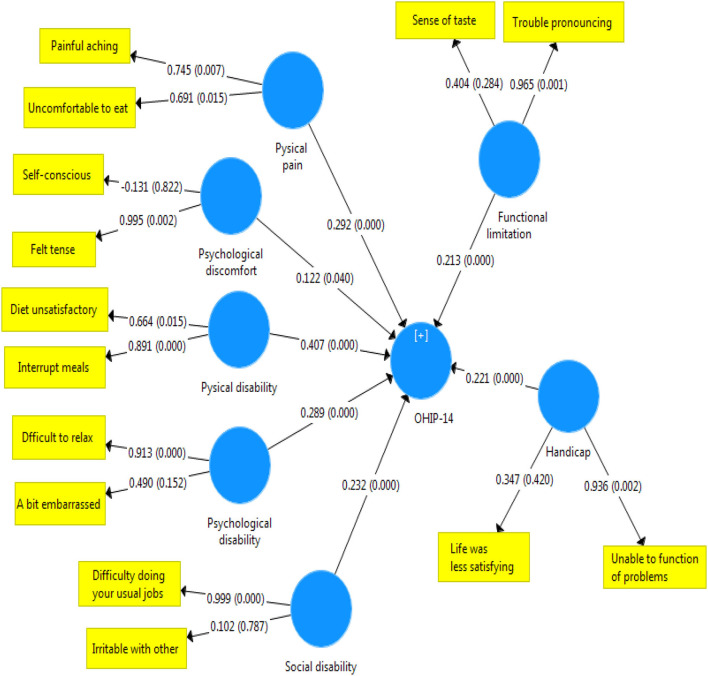
Status and severity of the relationship between scores in the dimensions of OHIP-14 scale and the total score of quality of life related to oral health in narcotic and stimulant users.

Later, intensity of the relationship was examined between the dimensions of oral health-related quality of life and the overall score of OHIP-14 in terms of the substance type. Figures 1–3 illustrate the theoretical model obtained.

## Discussion

The present study aimed at evaluating the quality of life related to oral health in patients who referred to MMT centers. The findings showed that the quality of life associated with oral health was significantly lower in people who use stimulants than narcotics. Although this rate of discrepancy was not severe, a significant difference was observed between scores of the two groups of drug abusers in the areas of mental disability, mental distress, and physical disability. More specifically, the effect of oral problems caused by using stimulants was higher on dimensions of quality of life, such as distraction, stress, peace disturbance, confusion in group, life dissatisfaction, and inability to perform activities. In other words, stimulants affect mental health and quality of life related to oral health negatively. Consistent with these findings, Nazemi et al. (22) reported that different dimensions of mental health (anxiety and social dysfunction) were significantly associated with the OHIP-14 index. Our findings on Figure 1 also corroborate this point. According to Figure 2, in addicts who use narcotics, physical disability was most associated with quality of life related to health. These results confirm the findings reported in the literature indicating that the type of substances applied affect the status of oral health indicators (23). This finding can be justified by mentioning that the most common oral lesions caused by using stimulants include dry mouth (16, 24, 25), spongy gingiva, and erythematosus with margin (16) that plays the most important role in the decline of their oral health (16). Islami et al. showed that the means of ruxism, rampant carries, xerostomia, attrition, and DMFT were higher in people with a history of using amphetamines stimulants (25). In contrast, missing teeth and caries had a significant relationship with drug abuse (26).

Generalized pigmentation was observed only in methamphetamine users. According to the literature, the duration of drug withdrawal was significantly associated with the incidence of oral lesions, so that less pigmented lesions were observed in addicts who have quit their addiction even for 1 month. In other words, increased duration of withdrawal improved the pink color, firmness, and stippled knife edge margin of the gum (16). Eslami et al. (25) conducted a study in Tabriz noting that abuse of stimulants (amphetamines and methamphetamines) was higher than narcotics (opium syrup) in women. This pattern change can explain higher incidence of oral health-related psychological problems followed by using stimulants. In this regard, many other factors may be influential, including the effect of addiction treatment methods, such as methadone therapy and withdrawal duration (16). A study was investigated amphetamine-dependent people and found that they intook more sugary foods, crumbs (instead of main meals), and drank cranberry drinks compared with the healthy individuals. Drug addicts believed that substance use reduced their appetite and increased their cravings for sugar (27). In the present study, only one fourth of those who referred to the substance abuse centers had been visited by dental professionals during the past year and a significant percentage of participants reported lack of a history of visits to dentists over the last 3 years. In Boston, about half of men and women who were addicted to drugs had more than a year since their last dental visit or were unable to recall the time of their last visit (28). According to the results of other studies, it seems that referring to dentists to receive educational and medical services is low among drug addicts around the world.

By comparing the results of this study with those reported in the literature, it can be claimed that the type of substance used by addicts is probably a more decisive factor than other personal variables in terms of the quality of life related to oral health. This finding can be justified by mentioning that quality of life related to oral health showed no significant difference not only among different levels of age, education, employment, economic status, marriage, and chronic illness, but also in other underlying factors such as a history of dental visits or the use of toothbrushes. Such discrepancy in the findings may be attributed to the sample size or the participants' characteristics since they were selected among patients who referred to MMT centers through available sampling method. Therefore, findings of the study are not generalizable to the population. In the studies by Shekarchizadeh et al. (8), Mahnaz Heydari (29), Ahmadi et al. (17), Esfahanizadeh (28), and Darvishpour Kakhki et al. (30) a significant relationship was found between the quality of health-related life and factors such as education, age, gender, and teeth condition (8, 29–31). This finding is in contrary to the findings of the present study. Given that the participants of the above-mentioned studies included women, retired, or elderlies of different cities, differences in the results can be attributed to diversity in their nutritional, environmental, cultural, or genetic factors. However, results of the studies by Akbari (13), Khabazian (32), Gholami (31), and Khatmi Nasab (33) are consistent with our findings in reporting that age and education had no relationship with quality of life related to oral health (13, 31–34). In this study, the total score of the oral health-related quality of life was 34.89 ± 6.50. Considering that these mean scores were received from the addicts who referred to addiction treatment centers, our findings cannot be representative of all addicts in Ahvaz. Moreover, substance use rates are naturally higher among the large population of addicts who are homeless and sleep on the street. As a result, future researchers are recommended to investigate addicts who are not in medical centers to provide a more comprehensive viewpoint in this area.

### Limitations of the Study

Due to the cross-sectional nature of this study, some limitations existed in reporting the definitive causal relationship between the studied variables. Furthermore, this study did not investigate addicts under treatment due to specific problems in accessing them.

## Conclusion

Based on the findings, the quality of life index related to oral health is more unfavorable in stimulant users than narcotic users. Therefore, authorities are recommended to focus their interventions and research programs to improve health-related quality of life in addicts, especially stimulant users.

## Data Availability Statement

The original contributions presented in the study are included in the article/supplementary material, further inquiries can be directed to the corresponding author/s.

## Ethics Statement

The studies involving human participants were reviewed and approved by Ahvaz Jundishapur University of Medical Sciences of Ethics Committee. Written informed consent for participation was not required for this study in accordance with the national legislation and the institutional requirements.

## Author Contributions

MC was principal investigators of the study. MC and HM were advisors of the study and performed the statistical analysis. FS was collected the data and drafted the manuscript. All authors contributed to the design, data analysis, assisted in the preparation of the final version of the manuscript, read, and approved the final version of the manuscript.

## Conflict of Interest

The authors declare that the research was conducted in the absence of any commercial or financial relationships that could be construed as a potential conflict of interest.

## Publisher's Note

All claims expressed in this article are solely those of the authors and do not necessarily represent those of their affiliated organizations, or those of the publisher, the editors and the reviewers. Any product that may be evaluated in this article, or claim that may be made by its manufacturer, is not guaranteed or endorsed by the publisher.

## References

[1] Chen CY Lin KM. Health consequences of illegal drug use. Curr Opin Psychiatry. (2009) 22:287–92. 10.1097/YCO.0b013e32832a2349 19378381

[2] Ma H Shi XC Hu DY Li X. The poor oral health status of former heroin users treated with methadone in a Chinese city. Med Sci Monit. (2012) 18:PH51–5. 10.12659/MSM.882611 22460103 PMC3560821

[3] Mokri A. Brief overview of the status of drug abuse in Iran. Arch Iran Med. (2002) 5:184–90.

[4] Brondani M Park PE. Methadone and oral health – a brief review. J Dent Hygiene. (2011) 85:92–8. 21619737

[5] Brienza RS Stein MD Chen M Gogineni A Sobota M Maksad J. Depression among needle exchange program and methadone maintenance clients. J Subst Abuse Treat. (2000) 18:331–7. 10.1016/S0740-5472(99)00084-7 10812305

[6] Terry D. Oral effect of drug abuse. Crit Rev Oral Biol Med. (1992) 3:163–84. 10.1177/10454411920030030101 1571470

[7] Sosnowski R Kulpa M Zietalewicz U Wolski JK Nowakowski R Bakuła R. Basic issues concerning health-related quality of life. Cent European J Urol. (2017) 70:206–211. 10.5173/ceju.2017.923 28721291 PMC5510334

[8] Shekarchizadeh H Khami MR Mohebbi SZ Ekhtiari H Virtanen JI. Oral health of drug abusers: a review of health effects and care. Iran J Public Health. (2013) 42:929–40. 26060654 PMC4453891

[9] Mary V Mahendra J John J Moses J Ebenezar A Kesavan R. Assessing quality of life using the oral health impact profile (OHIP-14) in subjects with and without orthodontic treatment need in Chennai, Tamil Nadu, India. J Clin Diagno Res. (2017):11:ZC78–ZC81. 10.7860/JCDR/2017/27309.10442 28969279 PMC5620926

[10] Pasiga BD Djamaluddin N Akbar FH. Oral health status and saliva characteristics of drug user at the rehabilitation center in Makassar. Sys Rev Pharmacy. (2020) 11:24–30.

[11] Lo Giudice G Cicciù M Polimeni A Lizio A Lo Giudice R Lauritano F. Oral and dental health of Italian drug addicted in methadone treatment. Oral Sci Int. (2019) 1:1–7. 10.1002/osi2.1000

[12] Hossain KMS Kakoli AS Mesbah FB Mian AH. Prevalence of oral and dental diseases and oral hygiene practices among illicit drug abusers. J Alcohol Drug Depend. (2018) 6:301.

[13] Akbari M. Evaluation of oral health status and dental need assessment in narcotic drug abusers. J Mash Dent Sch. (2015) 39:191–200. 10.22038/JMDS.2015.4798

[14] Kheirollahi Khatereh. Evaluation of oral health indices in people referring to outpatient addiction treatment centers in Yazd in 2019. J Res Dent Sci. (2020) 17:219–28. 10.52547/jrds.17.3.219

[15] Ghane Mehrdad. Oral health behavior of in-treatment female drug addicts in Tehran. Med-Tehran Univ Med Sci. (2016) 29:60–9. Available online at: http://jdm.tums.ac.ir/article-1-5476-en.html32141600

[16] Sadri D Jolehar M Salehian S Hajmostafazadeh A. A survey on the pattern of consumption and oral manifestations of a group of addicts in Tehran in 2017. J Tehran Univ j Res Dent Sci. 2019:16:234. 10.29252/jrds.16.3.234

[17] Ahmadi A Sahaf R Rashedi V Akbari Kamrani AA Shati M Delbari A. relationship between oral health and demographic characteristics in retired elderly people in Iran (Persian). Iranian J Ageing. (2019) 13:452–63. 10.32598/SIJA.13.4.452

[18] Jamshidi F Nazari I Malayeri HT Rahimi Z Cheraghi M. Pattern of drug abuse in addicts self-referred drug rehabilitation centers in Khuzestan province - Iran, 2014–2015. Archiwum medycyny sadowej i kryminologii. (2016) 66:1–12. 10.5114/amsik.2016.62330 28155984

[19] Jamshidi F Nazari I Cheraghi M. Risky behaviors of injecting drug users (IDUs) referred to addiction rehabilitation centers in Khuzestan Province in 2014. J Health Allied Sci. (2017) 16:5–7. Available online at: http://www.ojhas.org/issue62/2017-2-5

[20] Motallebnejad M Noghani A Tamaddon A Khafri S. Assessment of oral health status and oral health–related quality of life in thalassemia major patients. J Mazandaran Univ Med Sci. (2014) (119):83–94. Available online at: http://jmums.mazums.ac.ir/article-1-4772-en.html

[21] Navabi N Nakhaee N Mirzadeh A. Validation of a persian of the oral health impact profile (OHIP-14). Iranian J Publ Health. (2010) 39:135–9. 23113047 PMC3481693

[22] Nazemi N Momeni H Bashardoost N Abrishami M. Evaluation of oral health and psychological health related quality of life in patient with temporomandibular disorders. J Isfahan Dent Sch. (2019) 15:138–47.

[23] O'Sullivan EM. Dental health of Irish alcohol/drug abuse treatment centre residents. Community Dent Health. (2012) 29:263–7. 23488206

[24] Smit DA Naidoo S. Methamphetamine abuse: oral symptoms and dental treatment needs. S Afr Dent J. (2016) 71:150–4.

[25] Eslami H Jafari Heidarloo M Pakdel F. Orodental manifestations in methamphetamine users refereeing to oral medicine department, and their dental considerations. J Urmia Univ Med Sci. (2014) 25:1–11. Available online at: http://umj.umsu.ac.ir/article-1-2097-en.html

[26] Mohammadi TM Hasheminejad N Salari HR Rostamizadeh MR Najafipour H. Association between tooth loss and opium addiction: results of a community-based study on 5,900 adult individuals in South East of Iran in 2015. J Int Soc Prevent Communit Dent. (2017) 7:186–90. 10.4103/jispcd.JISPCD_189_17 28852634 PMC5558252

[27] Avena NM Hoebel BG. A diet promoting sugar dependency causes behavioral cross-sensitization to a low dose of amphetamine. Neuroscience. (2021) 122:17–20. 10.1016/s0306-4522(03)00502-5 14596845

[28] Esfahanizadeh N Farajollahi S Hajmaleki Z Daneshparvar N. Evaluation of the periodontal status among the institutionalized Iranian elderly supervised by Behzisti Organization in Tehran (2011). J Res Dental Sci. (2013) 10:199–204.

[29] Mahnaz Heydari Nasim Esnaashari Hajar Shekarchizadeh. Evaluation of oral health-related quality of life among patients with malocclusion. J Res Dent Sci. (2019) 16:185–94. 10.29252/jrds.16.3.194

[30] Darvishpoor Kakhki A Abed saeedi Z Abbaszadeh A. Social participation, barriers, and related factors in older people in Tehran. J Health Promotion Manag. (2014) 3:65–73.

[31] Gholami L Shahryari R Ansari-Moghaddam A. Effect of non-surgical periodontal treatment on oral health related quality of life. J Mash Dent Sch. (2020) 44:157–65.24372439

[32] Khabazian A Azarnoosh F Sadeghi SM. Evaluation of the effect of non-surgical periodontal therapy on the quality of life associated with oral health in patients with periodontitis and gingivitis referred to periodontology department of Yazd dental school. JDM. (2020)2:63–71.

[33] Khatmi Nasab N Shamshiri M Zamani U. The study of oral health status and its related quality of life in elderly people supported by welfare organization in ardabil city. J Health Care. (2020) 21:308–18. 10.29252/jhc.21.4.308

[34] Laslett AM Dietze P Dwyer R. The oral health of streetrecruited injecting drug users: prevalence and correlates of problems. Addiction. (2008) 103:1821–5. 10.1111/j.1360-0443.2008.02339.x 19032532

